# Integrative multicohort analysis reveals consistent sex differences in gut microbiota of multiple sclerosis patients

**DOI:** 10.1128/msystems.00416-26

**Published:** 2026-07-22

**Authors:** Irene Soler-Sáez, Cristina Galiana-Roselló, Rubén Grillo-Risco, Gwen Falony, Vanja Tepavčević, Sara Vieira-Silva, Francisco García-García

**Affiliations:** 1 Computational Biomedicine Laboratory, Principe Felipe Research Center (CIPF) 16252, Valencia, Spain; 2 Host-Microbe Interactomics, Wageningen University & Research (WUR) 4508, Wageningen, the Netherlands; 3 Laboratory of Comparative and Regenerative Neurobiology, Cavanilles Institute of Biodiversity and Evolutionary Biology, University of Valencia 16781, Paterna, Spain; 4 Department of Cell Biology, Functional Biology and Physical Anthropology, University of Valencia 16781, Burjassot, Spain; 5 Systems Biology and Multiomics Research Group, IREC, UCLouvain, Brussels, Belgium; Istanbul Medipol Universitesi Uluslararasi Tip Fakultesi, Istanbul, Türkiye; Brigham and Women's Hospital, Boston, Massachusetts, USA

**Keywords:** multiple sclerosis, sex differences, human gut microbiome, meta-analysis, interstudy heterogeneity, *Eggerthella*, *Eisenbergiella*

## Abstract

Biological sex is a key determinant in the onset and progression of multiple diseases. In multiple sclerosis (MS), females exhibit higher disease prevalence, earlier onset, and more pronounced inflammatory activity, whereas males tend to experience a more severe neurodegenerative course, characterized by accelerated central nervous system damage and increased brain atrophy. The gut microbiome has emerged as a critical factor in MS, as its composition can either ameliorate or exacerbate disease progression. In this study, we aimed to identify reproducible sex-associated differences in gut microbial composition across independent cohorts of MS patients. Through a systematic search, we identified six independent studies based on 16S rRNA gene sequencing, comprising a total of 337 samples. Despite substantial interstudy variability, sex-associated differences were more pronounced in MS patients than in healthy controls. We identified 11 microbial taxa showing significant sex-associated differences in MS, nine enriched in females, and two in males. Notably, the female-enriched taxa *Eggerthella* and *Eisenbergiella* were associated with specific MS subtypes and higher disability. To facilitate the use of our findings by the scientific community, we developed a freely accessible web-based tool that provides full access to our results. Thus, in this work, we identified consistent and reproducible sex differences in the gut microbiota of MS patients, highlighting the importance of incorporating sex as a critical variable in microbiome research, with potential implications for understanding disease heterogeneity in MS.

**IMPORTANCE:**

Multiple sclerosis (MS) affects females and males differently, but the biological reasons behind these differences are not fully understood. One potential factor is the gut microbiome (i.e., the community of microorganisms living in our intestines), which can influence immune function and disease progression. In this study, we analyzed data from multiple independent cohorts and found consistent differences in gut microbial composition between female and male MS patients. Notably, certain bacteria were more abundant in females and were linked to more severe disease features. We also developed a freely accessible web tool where researchers can explore the complete findings in detail. Our results highlight the importance of considering sex as a key factor in microbiome research and may help guide more personalized approaches to understanding and treating MS.

## INTRODUCTION

Multiple sclerosis (MS) is a chronic immune-mediated neurodegenerative condition of the central nervous system (CNS). In this disease, peripheral immune cells trigger autoreactive responses against the myelin sheath, leading to the formation of focal plaques that cause structural and functional damage (1, 2). MS is a highly heterogeneous disease structured in distinct clinical courses—relapsing–remitting multiple sclerosis (RRMS), primary progressive multiple sclerosis (PPMS), and secondary progressive multiple sclerosis (SPMS)—that represent a dynamic continuum between inflammatory and neurodegenerative mechanisms (3).

The gut microbiota has recently emerged as an important contributor to MS pathogenesis. Studies in animal models have determined that the disease does not develop in model organisms lacking a gut microbial community (4, 5). Moreover, fecal microbiota transplantation from MS patients triggered disease progression in the experimental autoimmune encephalomyelitis (EAE) mouse model (6, 7). These effects are thought to be partly mediated by microbial-dependent T-cell regulation at the gut mucosal interface, extending to systemic-level immune alterations (8). The increased permeability of the gastrointestinal and blood–brain barriers associated with MS may facilitate the translocation of bacterial components that can influence the synthesis and maintenance of the myelin sheath (9, 10). In human cohorts, multiple investigations have reported alterations in the abundance of specific taxa when comparing gut microbiomes of patients with MS to healthy controls (6, 11–21). However, these findings have not been consistent across studies (22, 23), prompting efforts to integrate results across cohorts (24, 25).

MS heterogeneity is modulated by host factors such as sex, which strongly influences MS epidemiology and clinical outcomes (26). Female patients exhibit higher disease prevalence, younger age at onset, and more pronounced inflammatory activity. Meanwhile, male patients tend to suffer from more severe neurodegenerative progression characterized by accelerated CNS damage and increased brain atrophy (27–29). In light of these differences, distinct research approaches have explored and identified potential molecular and cellular mechanisms through which sex acts as a key biological determinant in MS pathophysiology (30–34). The contribution of sex to MS may extend beyond host-intrinsic mechanisms to include sex-specific differences in gut microbiota composition. Gut microbial communities display sex-associated differences across the human lifespan (35), being shaped by hormonal, immunological, and lifestyle-related factors that modulate host–microbiome interactions (36, 37). Sex-associated microbiota differences have been described in murine models of autoimmune diseases (e.g., type 1 diabetes [38] and primary biliary cholangitis [39]) and neurodegenerative disorders (e.g., Alzheimer’s disease [40] and Parkinson’s disease [41]). For Parkinson’s disease, this evidence has also been reported in human studies (42). Despite its description in other conditions, biological sex represents a major source of heterogeneity in MS that has not yet been systematically integrated into microbiome research.

Accordingly, this study aimed to identify sex-dependent differences in the gut microbiota composition of MS patients by integrating independent 16S rRNA sequencing data sets. By adopting a meta-analysis approach, we aimed to identify consistent sex-differential abundance patterns robust to interstudy heterogeneity. We further validated the identified patterns in the largest MS cohort to date (15) and examined whether sex-differential abundant genera are also associated with clinical features of MS, such as disease severity and its clinical course.

## RESULTS

### Systematic review and data set overview

**Fig 1:**
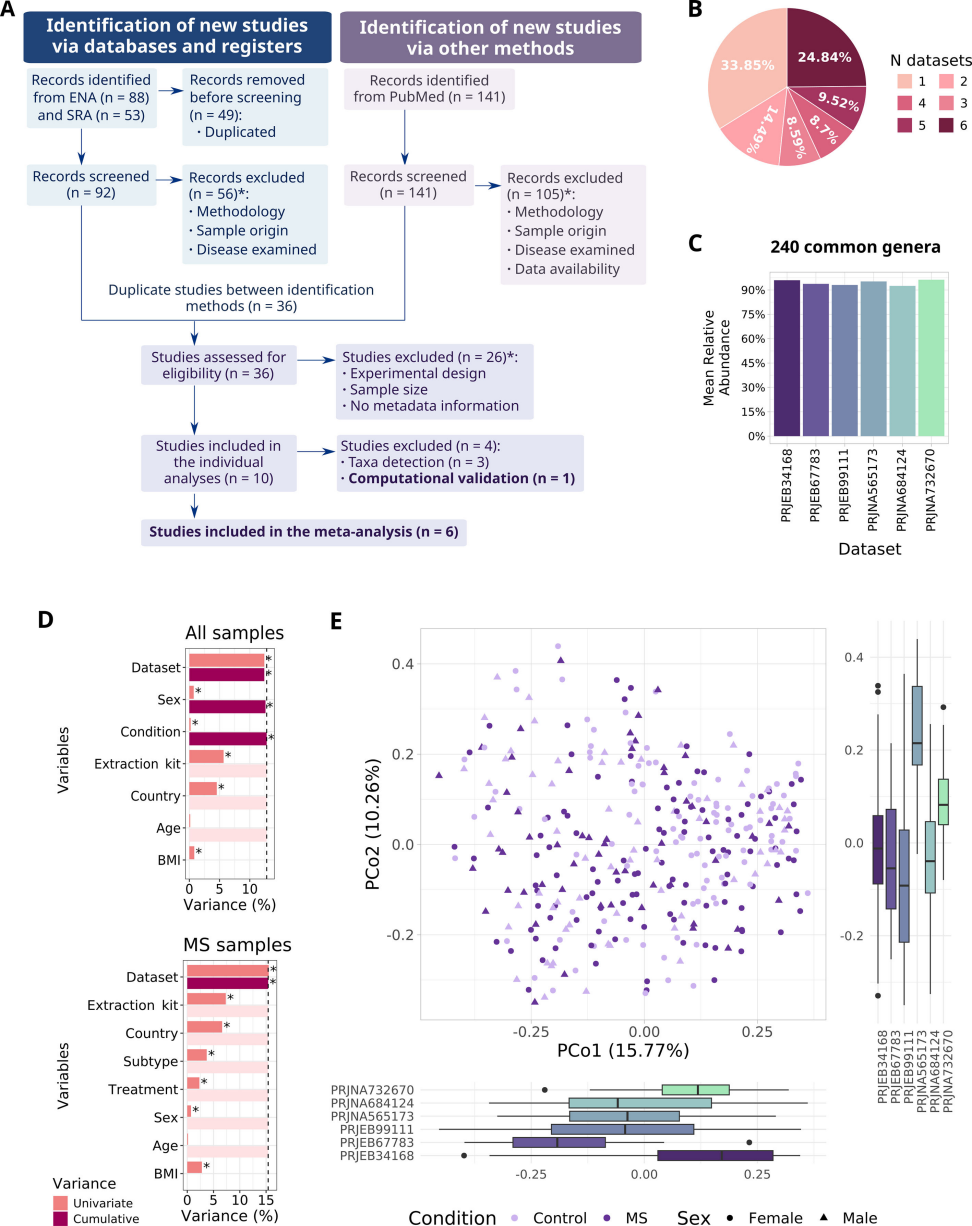
Overview of study selection and cross-data set characterization. (**A**) PRISMA flow diagram summarizing the results from the study selection process. *Studies have been excluded for one or more reasons simultaneously. (**B**) Percentage of taxa identified according to the number of studies in which they were detected. (**C**) Relative abundance of taxa shared across all included data sets. (**D**) Microbiome compositional variation explained by reported variables in all (top) and MS (bottom) samples, either individually (univariate) or in a multivariate model (cumulative). BMI was excluded from the multivariate model due to missing data in two studies (*N* missing data = 143). *, FDR < 0.05. Dashed lines denote total cumulative variance. (**E**) Principal coordinate sample distribution, colored by condition and sex, with study-specific distributions represented as box plots. BMI, body mass index; ENA, European Nucleotide Archive; FDR, false discovery rate; MS, multiple sclerosis; PCo, principal coordinate; PRISMA, Preferred Reporting Items for Systematic Reviews and Meta-Analyses; SRA, Sequence Read Archive*.*

Data search results are summarized in Fig. 1A. Ten studies met all predefined criteria for our analysis. Of these, we excluded three studies due to unexpected taxonomic composition (Fig. S1), and reserved one study for validation of the integrative approach. After sample-level quality control, we excluded 12 samples based on the criteria detailed in the Materials and Methods section. Thus, we included six studies in the meta-analysis, comprising a total of 337 samples. Table 1 depicts the characteristics of the final sample set for each study.

**TABLE 1:** Sample characteristics by data set included in the meta-analysis^*a*^

Characteristic	PRJNA684124	PRJNA732670	PRJEB99111	PRJEB34168	PRJNA565173	PRJEB67783
Sequencing platform	Illumina MiSeq	Illumina MiSeq	Illumina MiSeq	Illumina MiSeq	Illumina MiSeq	Illumina MiSeq
Sequencing read type	Paired end	Paired end	Single end	Paired end	Paired end	Paired end
16S hypervariable region(s)	V3–V4	V3–V4	V4	V4	V3–V4	V3–V4
Collection dates (years)	2020–2020	2017–2019	2013–2016	2013–2017	2015–2015	2021–2022
DNA extraction kit manufacturer	Qiagen	Qiagen	MoBio	MoBio	Qiagen	Biomes
Country	Italy	USA	USA	USA	Russia	Italy
*N* subjects	30	56	113	65	30	43
*N* controls (female:male)	15 (7:8)	34 (28:6)	53 (25:28)	39 (27:12)	15 (9:6)	12 (7:5)
*N* MS (female:male)	15 (11:4)	22 (17:5)	60 (39:21)	26 (22:4)	15 (6:9)	31 (19:12)
Age (mean ± SD)	60.67 ± 13.39	42.91 ± 11.68	43.19 ± 13.06	43.49 ± 12.18	40.3 ± 15.99	43.73 ± 14.20
BMI (mean ± SD)	Not reported	26.53 ± 6.08	Not reported	28.19 ± 5.86	23.46 ± 3.25	23.37 ± 3.79
MS subtype	RRMS	RRMS	RRMS	RRMS	PPMS	RRMS, SPMS
Treatment	Treated (*n* = 6) Untreated (*n* = 9)	Treated (*n* = 16) Untreated (*n* = 6)	Untreated (*n* = 60)	Untreated (*n* = 26)	Untreated (*n* = 15)	Treated (*n* = 30) Untreated (*n* = 1)
PMID	34206853	35472144	28893978	32743517	31888483	39402079

^*a*^ BMI, body mass index; MS, multiple sclerosis; PMID, PubMed identifier; PPMS, primary progressive multiple sclerosis; RRMS, relapsing–remitting multiple sclerosis; SD, standard deviation; SPMS, secondary progressive multiple sclerosis*.*

All studies recorded disease status (i.e., MS or control) and sex (i.e., female or male) information. Total samples were well balanced across conditions (168 controls and 169 MS samples), while the sex distribution skewed toward females (217 females and 120 males), consistent with the known sex-differential prevalence in MS.

The studies originated from diverse geographical regions. Age information was available for all of them, while the body mass index (BMI) was reported in four out of the six. The studies covered the three MS subtypes and included samples from treated and untreated individuals at the time of stool collection. All data were generated using Illumina MiSeq sequencing, targeting either the V4 or the V3–V4 regions of the 16S rRNA gene.

Across all samples, we identified 965 distinct genera, many of which were study and sample specific. Only 24.87% (240 genera) were consistently detected across all data sets (Fig. 1B). Importantly, these shared taxa accounted for more than 90% of the total relative abundance in every data set, with a mean relative abundance of 94.49% (Fig. 1C). Alpha diversity significantly differed across studies (Kruskal–Wallis test, *P* = 3.55 × 10⁻⁵), with lower diversity observed in data sets targeting the V4 region than those targeting V3–V4 (Fig. S2A). However, no significant differences were observed when stratifying samples by condition and sex (Kruskal–Wallis test, *P* = 0.21; Fig. S2B).

We next quantified the contribution of reported variables to microbiota compositional variation (Fig. 1D, top). All variables except “age” (adjusted *P* = 0.058) were significant in univariate models, with the data set variable being the main contributor to variation in microbial composition. In the multivariate model, data set, “condition” and “sex” remained significant, jointly explaining 12.8% of non-redundant variance. Consistently, principal coordinate analysis captured the main patterns of variation in microbiota composition (Fig. 1E). We next restricted the analysis to MS samples that incorporated disease-specific clinical features (Fig. 1D, bottom). The data set variable remained the dominant source of variation (*R*² = 12.4% across all samples and 15.5% in MS samples), with no multivariate model improving upon its explanatory power.

After identifying the data set variable as the main contributor to variation in the combined analysis, we explored potential sources of variability within each data set (Table S1). Most variables did not display significance. However, condition represented the most recurrent factor, reaching significance in three out of the six data sets, explaining approximately 1%–3% of the variance.

### Identification of sex-differential abundant genera in individual studies

Although the data set captured the major source of variation, condition and sex still emerged as significant contributors to host-related microbiome taxonomic variation. We next sought to identify sex-associated abundance patterns by condition within each data set, preserving between-data set variability for the subsequent meta-analysis.

**Fig 2:**
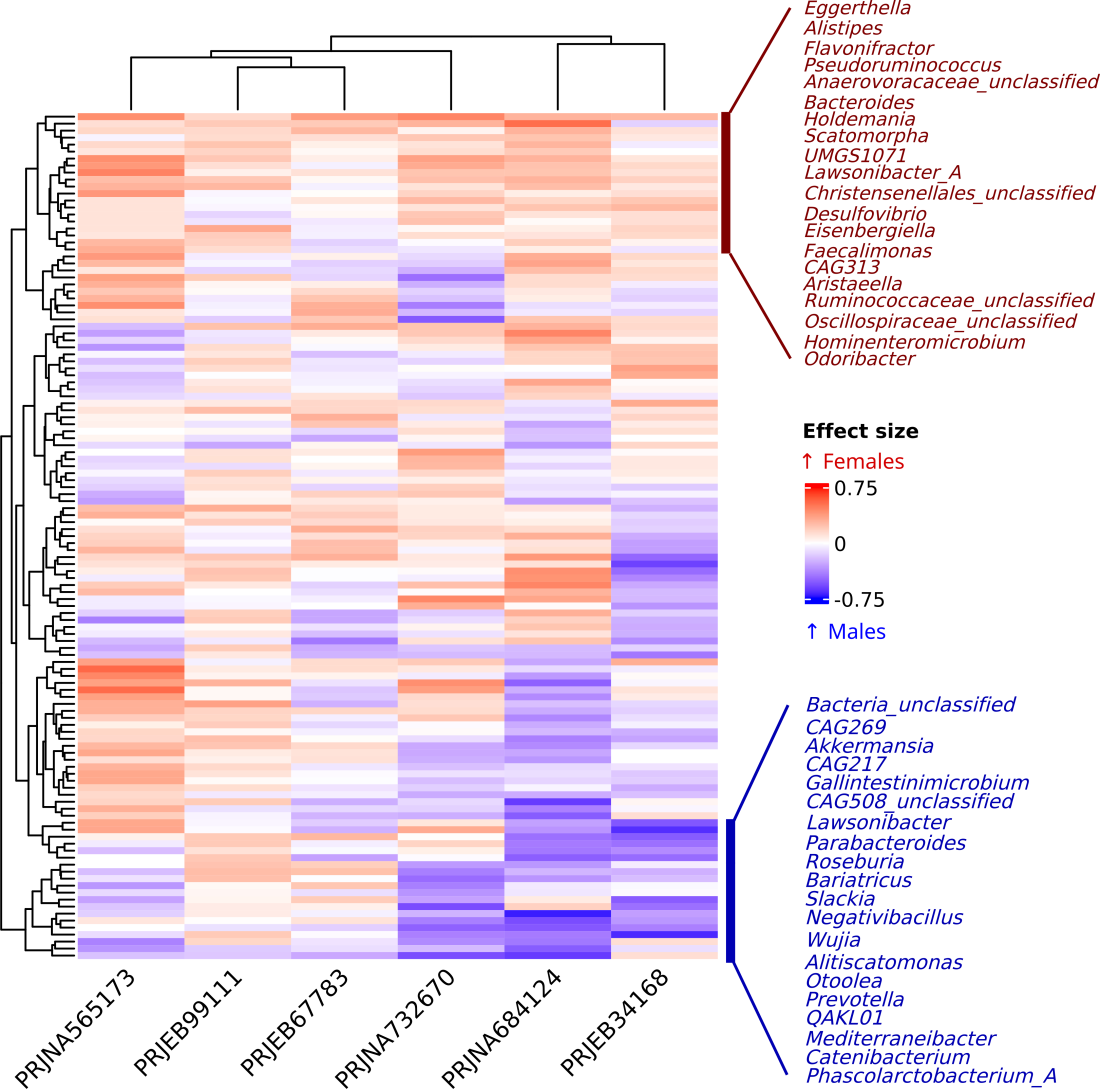
Sex-differential abundance patterns by individual data sets from the MS population. Columns (data sets) and rows (genera) clustered based on hierarchical clustering. The color indicates the direction of the effect size (red denotes higher abundance in females; blue denotes higher abundance in males). Clusters with the most sex-differential consistent patterns are highlighted: female enriched (red) and male enriched (blue).

Sex-differential patterns were assessed for controls (control females vs control males) and MS (MS females vs MS males) populations separately. Figure S3 provides complete results for both comparisons. We observed substantial heterogeneity in genus-level abundance patterns across data sets in both comparisons, with no taxa reaching significance after false discovery rate (FDR) correction (adjusted *P* < 0.05). In the control comparison, fewer taxa exhibited significant trends (*P* < 0.05, *n* = 17) than in the MS comparison (*P* < 0.05, *n* = 34), suggesting pathophysiology increases the gut microbiome sex differences. Meanwhile, sex-differential patterns in the MS comparison were qualitatively more consistent. Specifically, 40 taxa exhibited a tendency toward higher abundance in females (taxa highlighted in red) or in males (taxa highlighted in blue) in the majority of the data sets (Fig. 2).

Taken together, although the microbiome compositional data were heterogeneous across individual studies, different taxa—most notably in the MS comparison—exhibited consistent sex-differential patterns, potentially reflecting changes robust to study-specific technical and biological factors.

### Meta-analysis-based integration uncovered consistent sex-differential patterns in multiple sclerosis

To statistically assess the robustness of the sex-associated patterns observed in the individual data sets, we performed a random-effect meta-analysis for the controls and MS comparisons. No genera showed significant sex-differential abundance in healthy individuals, although eight exhibited significant trends prior to multiple testing correction (*P* < 0.05), with four more abundant in females (*Coprobacter*, *Agathobaculum*, *Eisenbergiella*, and *Evtepia*) and four more abundant in males (*Gemmiger*, *Limivivens*, *Slackia*, and *CAG217*).

**Fig 3:**
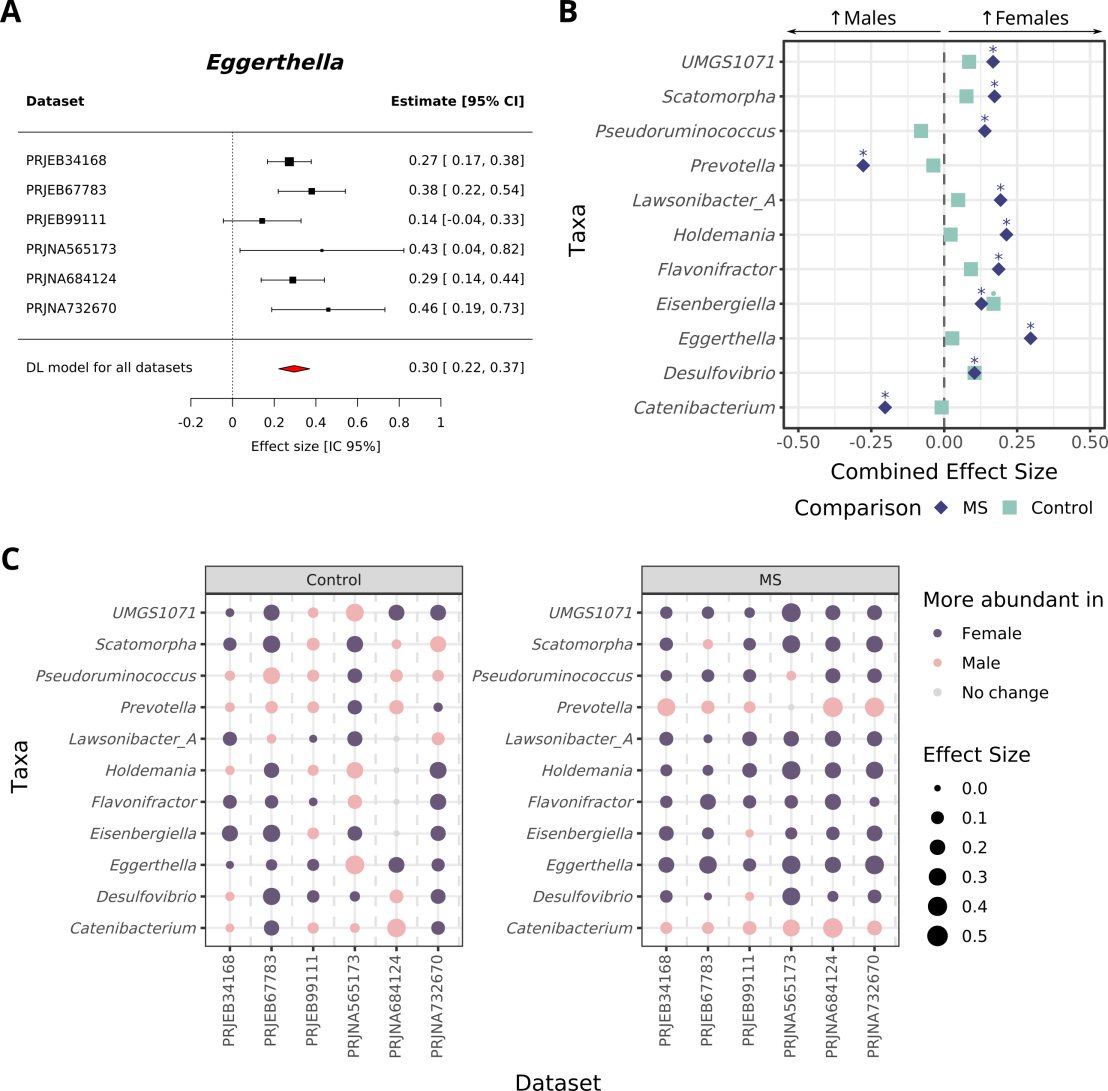
Integrative assessment of sex-associated microbial differences. (**A**) Forest plot for *Eggerthella* in the MS female vs MS male comparison. Squares represent individual effect sizes, and horizontal lines indicate the 95% confidence intervals. The red diamond denotes the combined effect size and its confidence interval. Positive values: higher abundance in MS females; negative values: higher abundance in MS males. (**B**) Sex-differential significant taxa in MS. The *x*-axis indicates the combined effect size of the comparison of females vs males in control (squares) and MS (diamonds) groups. *, adjusted *P* < 0.05; ⬤, *P* < 0.05. (**C**) Individual effect sizes of taxa identified as significant in the meta-analysis for control (left) and MS (right) comparisons. CI, confidence interval; DL, DerSimonian–Laird; MS, multiple sclerosis*.*

Strikingly, we observed 11 genera significantly differentially abundant in the MS comparison after FDR correction (adjusted *P* < 0.05), with nine genera more abundant in females (*Desulfovibrio*, *Eggerthella*, *Eisenbergiella*, *Flavonifractor*, *Holdemania*, *Lawsonibacter*, *Pseudoruminococcus*, *Scatomorpha*, and *UMGS1071*) and two more abundant in males (*Catenibacterium* and *Prevotella*). Influence analyses revealed that no more than two of the six studies substantially contribute to the combined effect size (i.e., the majority of studies contribute in a balanced manner to the consensus effect size and did not show evidence of publication bias or small-study effects). Funnel plots displayed the expected symmetric, inverted funnel shape. Together, these results support the reliability of the meta-analysis outcomes. As an illustrative example, we report the result for *Eggerthella* in Fig. 3A, along with the corresponding influence and funnel plots in Fig. S4A. Figure S5 to S7 provide the results for the remaining 10 significant genera.

The significant differences identified in the MS comparison were mainly sex-associated patterns already present in healthy individuals, rather than opposite sex-specific trends between health and disease states (Fig. 3B). Thus, the majority of taxa showed effect sizes with the same direction in the controls as in the MS comparison, although with smaller magnitudes in the former, with both greater heterogeneity across individual studies and overall weaker effects (Fig. 3C, left). By contrast, the MS comparison showed more consistent patterns with larger effect sizes across data sets, with 6 of the 11 taxa displaying concordant effects in all studies and the remaining in 5 out of the 6 (Fig. 3C, right). Both the heterogeneity in the control comparison and the consistency of results in the MS comparisons suggest that MS pathophysiology increases sex differences in gut microbiome composition.

### Validation of the identified genera in an independent cohort

To validate the findings, we analyzed the data from the iMSMS cohort (15) retrieved from the PRJEB32762 data set. Technical and biological characteristics of this data set are summarized in Table 2. This study was selected as the validation cohort due to its large sample size (*n* = 1,152) and the inclusion of participants from multiple geographical regions.

**TABLE 2:** Summary of demographic, clinical, and technical characteristics of the validation data set^*a*^

Variable	Total (*n* = 1,152)
Enrollment group	1 (*n* = 256, recruited before October 2016), 2 (*n* = 896, recruited before January 2019)
Sequencing platform	Illumina MiSeq
Sequencing read type	Paired end
16S hypervariable region(s)	V4
Collection dates (years)	2015–2019
DNA extraction kit manufacturer	Qiagen
Recruitment site	San Francisco (*n* = 328), Boston (*n* = 84), New York (*n* = 118), Pittsburgh (*n* = 24), Buenos Aires (*n* = 258), Edinburgh (*n* = 262), San Sebastián (*n* = 78)
*N* controls (female:male)	201:375
*N* MS (female:male)	400:176
Age (mean ± SD)	49.73 ± 12.53
BMI (mean ± SD)	26.11 ± 5.28
MS subtype	RRMS (*n* = 437), PPMS (*n* = 71), SPMS (*n* = 68)
Treatment	Treated (*n* = 367), untreated (*n* = 209)
MS duration years (mean ± SD)	14.21 ± 11.13 (not available = 1)
EDSS (mean ± SD)	2.6 ± 2.24
PMID	36113426

^*a*^ The number of samples per category is shown for categorical variables. The mean value was determined for numerical variables. BMI, body mass index; EDSS, expanded disability status scale; MS, multiple sclerosis; PMID, PubMed identifier; PPMS, primary progressive MS; RRMS, relapsing–remitting MS; SD, standard deviation; SPMS, secondary progressive MS*.*

**Fig 4:**
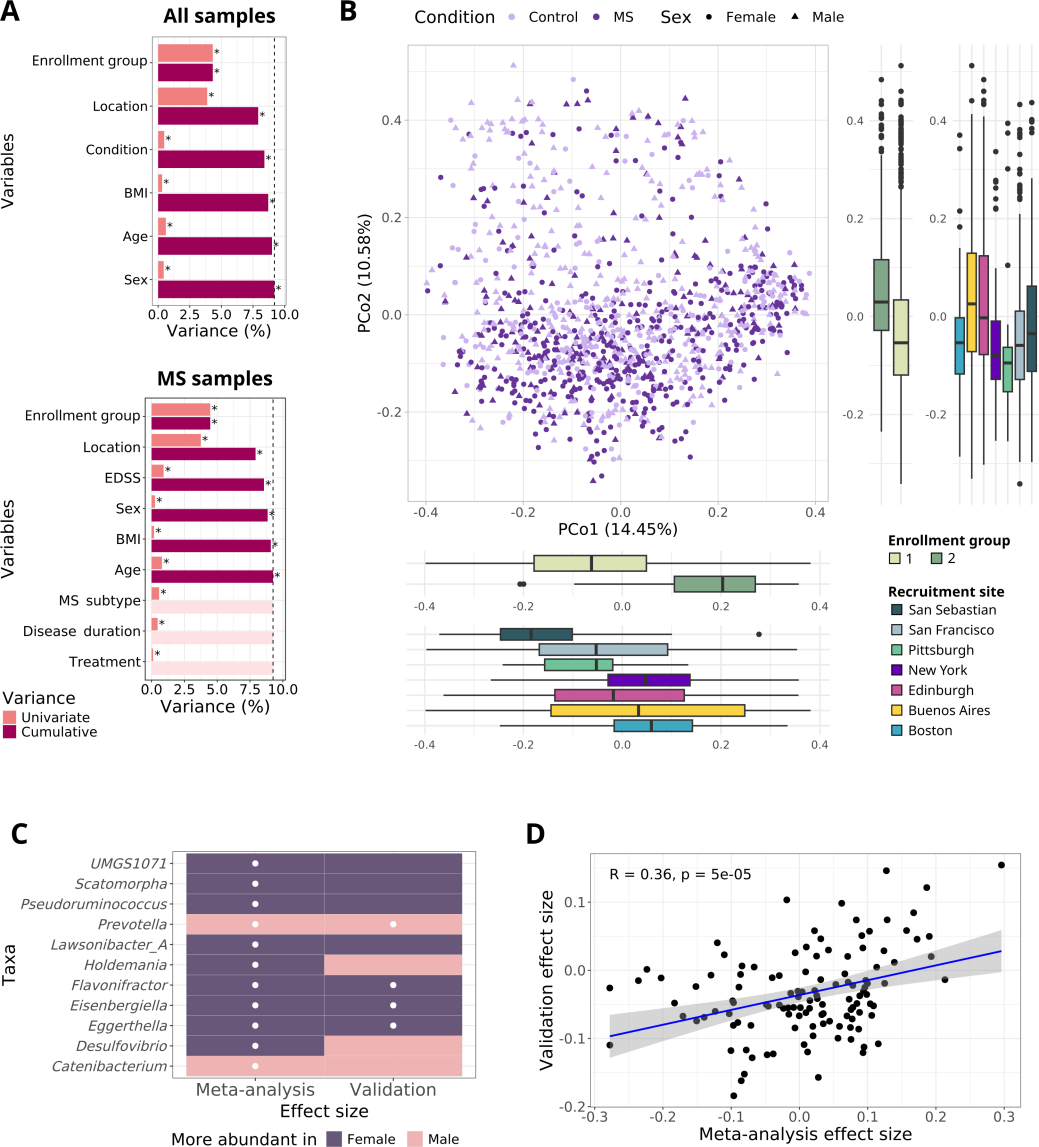
Characterization of the validation cohort and concordance of sex-differential abundance patterns in the MS comparison (MS females vs MS males). (**A**) Microbiome compositional variation explained by reported variables in all (top) and MS (bottom) samples, either individually (univariate) or in a multivariate model (cumulative). *, adjusted *P* < 0.05. Dashed lines denote total cumulative variance. (**B**) Principal coordinate sample distribution, colored by condition and sex. Enrollment group- and recruitment site-specific distributions are represented as box plots. (**C**) Direction of effect (higher abundance in MS females vs MS males) for taxa identified as significant in the meta-analysis, compared with corresponding results in the validation cohort. White dots denote adjusted *P* < 0.05. (**D**) Pearson correlation between the combined effect sizes from the meta-analysis and those obtained in the validation cohort. The blue line denotes linear regression fit; shaded areas indicate standard errors. BMI, body mass index; EDSS, expanded disability status scale; MS, multiple sclerosis*.*

The technical variables “enrollment group” and “recruitment site” constituted the largest known contributors to the microbial composition (Fig. 4A and B). The recruitment site was also associated with significant differences in alpha diversity (Fig. S8A and B), in agreement with the results from the original publication (15). Therefore, we accounted for both variables in the differential abundance analyses. Beyond technical factors, MS males presented a higher alpha diversity (Fig. S8C). The host-related biological variables recorded in this cohort—including condition, BMI, age, and sex—were incorporated into the multivariate model, together explaining 9.19% of the total variance. For MS samples, the cohort and recruitment site remained the dominant contributors to variability. In this subset, these two variables, together with the expanded disability status scale (EDSS), sex, and BMI, explained a total of 9.21% of non-redundant variance (Fig. 4A).

The significant sex differences identified in the meta-analysis approach were mostly replicated, with 82% of the taxa (9 out of 11) showing concordant directionality between the meta-analysis and the validation data set (Fig. 4C). Among these nine, four taxa reached statistical significance in the validation cohort even after FDR correction; namely, *Eggerthella*, *Eisenbergiella*, and *Flavonifractor* were more abundant in MS females, whereas *Prevotella* was more abundant in MS males. Correlating the effect sizes of the sex associations identified from the meta-analysis consensus with the results obtained in the validation cohort resulted in a positive correlation (ρ = 0.36, *P* < 0.05) (Fig. 4D), supporting the reproducibility of sex-associated gut microbiome patterns in MS despite substantial interstudy variability.

We also leveraged the whole-genome shotgun (WGS) sequencing data from the iMSMS cohort. Of the 11 taxa identified as significant in the MS comparison, the directionality of effect size was concordant with the one observed in the 16S rRNA gene sequencing validation for the majority of the evaluated taxa. Specifically, *Eggerthella* and *Flavonifractor* presented significantly higher abundance in MS females, whereas *Prevotella* was more abundant in MS males, supporting the reproducibility of the results beyond 16S rRNA sequencing resolution (Fig. S9).

To further characterize whether the observed sex differences were specific to MS, we applied linear mixed models exploring the interaction between sex and condition for the abundance of the 11 genera in the iMSMS 16S validation cohort (Table S2). Interestingly, *Eggerthella* and *Flavonifractor* showed significant effects for sex being enriched in females, but not for condition nor the interaction term, suggesting a generalized sex effect on the gut microbiota independent of disease status. A similar trend was observed for *Prevotella*, which was enriched in males regardless of condition, although not reaching statistical significance (*P* = 0.01). In contrast, *Eisenbergiella* presented a more complex pattern, with a significant effect for condition higher in MS (*P* = 0.03) and a trend toward higher abundance in females (*P* = 0.07), though the interaction term did not reach statistical significance (*P* = 0.15), suggesting a potential disease-modulated sex effect.

### The sex-differentially abundant genera *Eggerthella* and *Eisenbergiella* are associated with MS clinical features

We next assessed whether the four validated sex-differential abundant genera with strongest statistical support were associated with MS clinical features using the phenotypic information from the iMSMS cohort from the PRJEB32762 data set (validation data set), including MS subtype, EDSS, disease duration, and current treatment. Significant associations were identified for the female-associated *Eggerthella* and *Eisenbergiella* but not for *Flavonifractor* nor the male-associated *Prevotella*.

**Fig 5:**
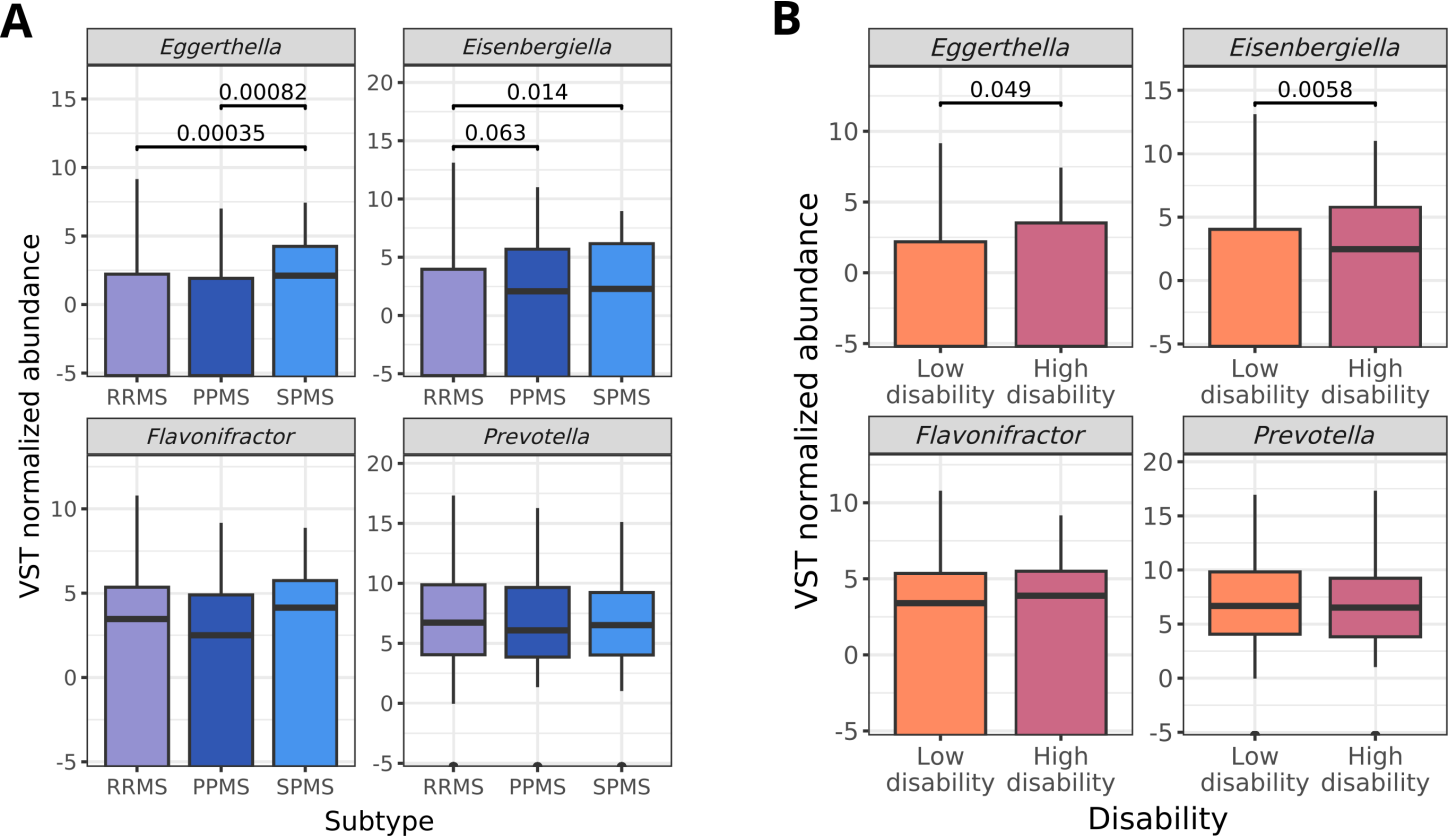
Normalized abundance of sex-associated taxa in multiple sclerosis stratified by (**A**) disease subtype and (**B**) degree of disability. Values indicate adjusted *P* values (**A**) from Dunn’s *post hoc* test (after *P* < 0.05 in Kruskal–Wallis test) and (**B**) Wilcoxon rank-sum test. PPMS, primary progressive multiple sclerosis; RRMS, relapsing–remitting multiple sclerosis; SPMS, secondary progressive multiple sclerosis; VST, variance stabilizing transformation*.*

*Eggerthella* was identified as more abundant in the SPMS subtype, corresponding to a more advanced disease stage after RRMS (Fig. 5A). Additionally, its abundance was slightly positively associated with disease duration (ρ = 0.10, *P* = 0.01) and age (ρ = 0.10, *P* = 0.01). As disease duration and age were themselves moderately correlated in MS samples (ρ = 0.57, *P* < 2.2e−16), we evaluated whether this age association reflected disease-related effects. Notably, we observed no association between *Eggerthella* abundance and age in the control group (ρ = 0.04, *P* = 0.33), suggesting that the observed associations could be linked to MS-related processes rather than to age alone.

Conversely, *Eisenbergiella* was found to be more abundant in progressive forms of multiple sclerosis (PPMS and SPMS) than in RRMS (Fig. 5A). Its abundance was slightly positively correlated with disease duration (ρ = 0.09, *P* = 0.03) and age (ρ = 0.13, *P* = 1.4 × 10^−^³). Unlike *Eggerthella*, the age association was replicated in the control cohort (ρ = 0.14, *P* = 8.3 × 10^−^⁴), suggesting that age-related changes in *Eisenbergiella* are not MS specific. Higher *Eisenbergiella* abundance was observed in patients with greater disability (EDSS > 4) relative to those with mild and low-grade disease forms (Fig. 5B). However, patients with higher disability were significantly older (Wilcoxon test, *P* < 2.2e−16), suggesting its role in MS could be confounded by age.

*Eggerthella* and *Eisenbergiella* exhibited associations with different MS features. They were not consistently detected together across MS samples. *Eggerthella* was detected in 62.5% of samples, while *Eisenbergiella* was identified in 54%, both taxa being simultaneously detected in 23.5% of samples. In cases where both taxa were present, their abundances were positively correlated (ρ = 0.32, *P* < 0.0018), suggesting similar fitness responses to host-related factors.

None of the four validated genera showed significant differences in abundance between treated and untreated groups (Fig. S10), suggesting that treatment effects do not confound the observed associations. Furthermore, no clinical or demographic variable examined exhibited sex-specific differences (i.e., they present similar distributions in females and males) (Fig. S11).

### Web-based resource for interactive exploration of results

**Fig 6:**
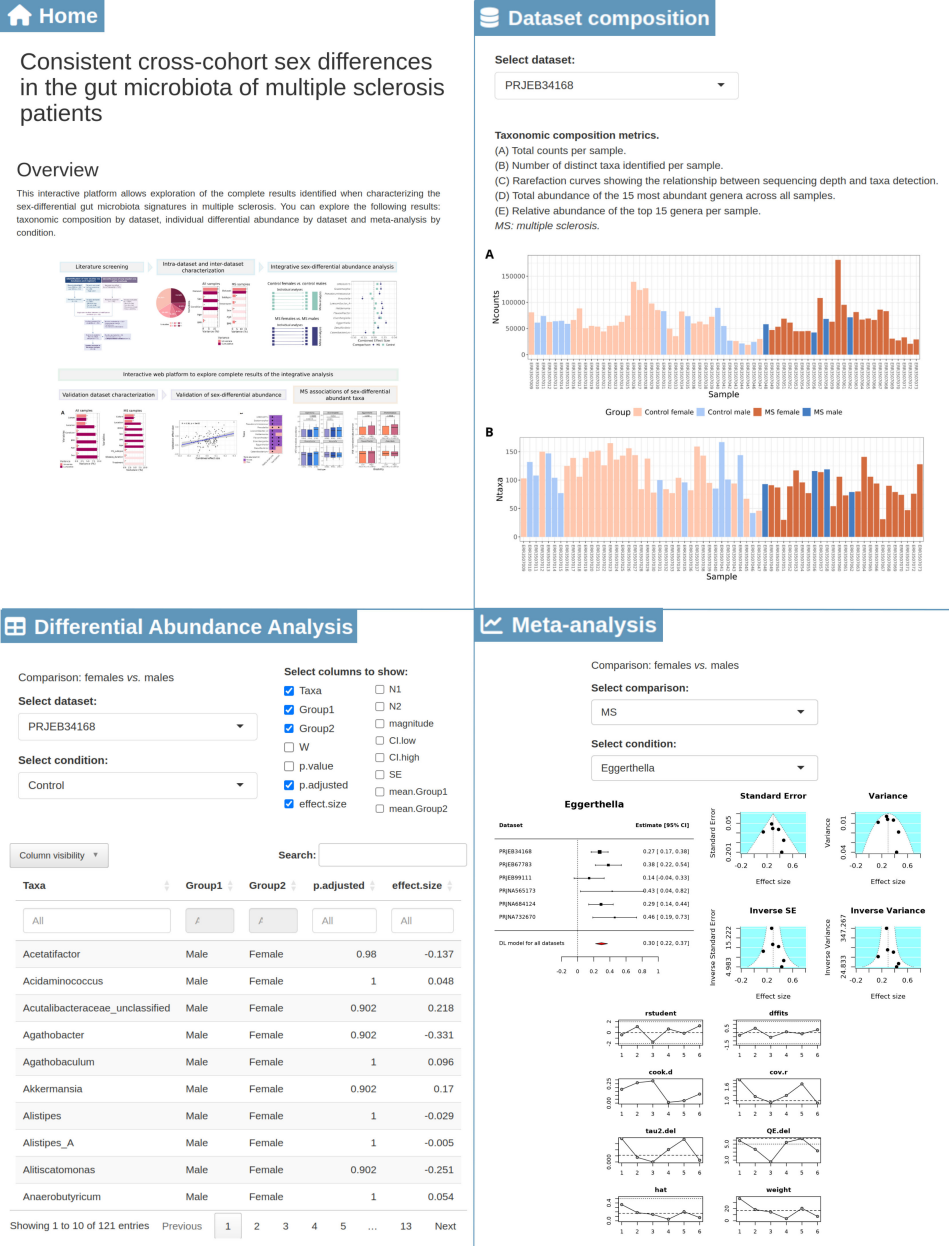
Overview of the interactive web platform. The web resource comprises four tabs: “home,” summarizing the study design; “data set composition*,”* reporting taxonomic composition metrics; “differential abundance analysis,” presenting sex-differential results in control and MS populations for each data set; and “meta-analysis,” providing forest, funnel, and influence plots, along with summary tables for each taxon.

The complete findings from this study are available through a publicly accessible web resource (https://irsoler.shinyapps.io/metaanalisis_16S_MS/) (Fig. 6), which provides an interactive interface organized into four tabs. The “home” tab presents a graphical overview of the study design and objectives. The “data set composition” tab displays the taxonomic composition for each sample, including total counts, the number of unique taxa, rarefaction curves, and the total and relative abundance of the 15 most abundant taxa per data set. The “differential abundance analysis” tab allows users to explore sex-associated differential abundance results for each data set in control and MS populations, enabling examination of metrics for specific genera and application of user-defined filters. Finally, the “meta-analysis” tab provides interactive access to forest, funnel, and influence plots for selected genera and comparisons, along with summary tables containing the corresponding results.

## DISCUSSION

The involvement of the gut microbiota in MS pathogenesis is well established, as this disease does not develop in germ-free mouse models (4, 5), and distinct taxa have been linked to MS in human studies (24, 25). Likewise, sex is known to influence both disease prevalence and progression (26), yet sex-associated differences in gut microbiota composition in MS remain poorly characterized.

In this study, we applied an integrative approach analyzing 16S rRNA gene sequencing data sets to investigate sex-associated differences in the gut microbiota of MS patients. We observed substantial variability across studies, with the data set of origin identified as the main source of variation (dbRDA analysis, variance >10%). Such heterogeneity has been previously reported, as technical differences including DNA extraction methods and targeted 16S rRNA gene regions can affect the microbial composition of the data set (43–45). Beyond technical factors, this data set-driven variability may also reflect biological heterogeneity among individuals and environmental factors (46). Specifically in MS, research findings have been discordant across studies identifying taxa that differ between MS patients and healthy controls. To overcome this, systematic reviews (22, 23) and meta-analyses (24, 25) have sought to identify consensus patterns.

Within this context, we conducted sex-differential abundance analyses to characterize data set-specific shifts. The results were then integrated using a random-effect meta-analysis strategy. Sex-associated differences in MS (i.e., MS females vs MS males) were more pronounced and consistent than in controls (i.e., control females vs control males). Particularly, we identified 11 taxa with significant differences in MS, 9 more abundant in females and 2 more abundant in males. These findings were validated using the iMSMS consortium data set (15), the largest and most geographically diverse cohort identified in our systematic review. Overall, 9 of the 11 patterns were replicated, and 4 remained significant after multiple testing correction: *Eggerthella*, *Eisenbergiella*, and *Flavonifractor* were more abundant in MS females, whereas *Prevotella* was more abundant in MS males. Notably, *Eggerthella* and *Eisenbergiella* were also associated with specific MS subtypes and higher disability degrees.

*Eggerthella* has been associated with pro-inflammatory environments in both mouse models (47) and humans (48). It has been quantitatively linked to the human enterotype associated with systemic inflammation (Bact2), increasing in abundance in overall lower microbial load fecal microbiota (49). Moreover, Chen et al. (49) identified *Eggerthella* as one of the top taxa enriched in rheumatoid arthritis, where this bacteria may exacerbate disease severity. The connection of *Eggerthella* to immune-mediated diseases may involve the activation of Th17 cells (50, 51), which play a pivotal role in MS pathogenesis to trigger autoimmunity (52). Indeed, *Eggerthella* has been reported to be more abundant in MS patients compared with healthy controls (6). Our observations further suggest a higher abundance in females, which may reflect either its contribution to the enhanced inflammation observed in females vs. males or its fitness advantage in the autoimmune context. We additionally found that *Eggerthella* abundance was positively associated with disease duration—but not with age in controls—and with the SPMS subtype, suggesting a potential link with MS chronicity.

*Eisenbergiella* was first reported in 2014 as a genus with the capacity for short-chain fatty acid production (53). *Eisenbergiella* has been reported to be enriched in diverse conditions, like gestational diabetes mellitus (54), end-stage renal disease (55), colorectal cancer (56), and the autoimmune disease systemic lupus erythematosus (57). It was also associated with neurodegenerative diseases, reported as potentially protective in Alzheimer’s disease (58) but enriched in Parkinson’s disease (59). In MS-based studies, it was identified as more abundant when compared with healthy controls, both in human cohorts and in EAE models (15, 60). Yoon et al. (60) also reported that ileal microbiota derived from patients with MS, but not from their healthy monozygotic twins, induced MS-like disease in germ-free transgenic mice. Within this context, *Eisenbergiella* emerged as a candidate disease-associated taxon, suggesting a potential contributory role in MS pathogenesis, to which we now add a sex-differential role being female enriched. We also observed a positive correlation with age in MS and controls, consistent with previous reports linking *Eisenbergiella* abundance to long-lived individuals (61).

Of note, in the iMSMS publication (15), the authors compared the gut microbiome composition of MS patients and control individuals, adjusting for sex among other covariates. They identified *Eisenbergiella* as an MS-associated taxon with higher abundance in progressive MS, consistent with our findings. In contrast, *Eggerthella* was not identified as significantly associated with MS. In our study, the abundance of *Eggerthella* appears to be primarily determined by sex rather than by disease condition itself, suggesting that its differential abundance may reflect a sex-dependent modulation exacerbated within the MS context rather than a direct disease-specific association. These findings highlight the complementary value of condition-stratified sex-associated analyses in identifying differential abundance patterns that may remain undetected in case–control study designs.

Although we did not identify direct associations between these taxa and MS clinical features, *Flavonifractor* and *Prevotella* are known to modulate the host immune system. *Flavonifractor* was identified as more abundant in females with MS compared with males with MS. This genus is involved in the metabolism of dietary flavonoids, bioactive compounds derived from fruits and vegetables (62). *Flavonifractor* has been associated with colorectal cancer, authors suggesting a potential role for its metabolic ability to degrade anticarcinogenic flavonoids (63). However, experimental studies in mice suggest a protective role, contributing to the attenuation of autoimmune responses (64, 65) and to improved responses to CAR-T therapy (66).

*Prevotella* was the single validated taxon more abundant in males than in females with MS. This genus has been linked to diets rich in fiber and complex carbohydrates (67). Of note, in the meta-analysis from Lin et al. (24), this taxon was reported to be decreased in MS patients compared with controls across all included studies, reaching statistical significance in more than half. *Prevotella* has been shown to suppress Th17-mediated autoimmune responses and to reduce disease severity in EAE (68). Nevertheless, it has also been associated with chronic inflammation (69), suggesting that its immunomodulatory role is highly context dependent. Our results suggest that studying the role of these taxa in the pathophysiology of MS using experimental models should be done with sex effects kept in mind.

To the best of our knowledge, this article describes the first integrative study that explicitly evaluates the influence of sex in MS within the context of the human gut microbiome. Some of the identified taxa had previously been associated with MS without considering the sex differences. Notably, some of the validated taxa show comparable sex-associated patterns in other diseases, such as *Eggerthella*, which has been reported to be more abundant in females in metabolic syndrome (70) and bronchial asthma (71). These convergent observations suggest the existence of shared, sex-related mechanisms across conditions. In addition, we identified microbial associations with clinical characteristics of MS, contributing to the characterization of how sex-specific microbiota composition may relate to disease course.

Limitations in this study should be acknowledged. Heterogeneity across cohorts, including differences in sample size, sequencing platforms, and targeted 16S rRNA regions, represents an important source of variability that may limit the generalizability of our findings. Limited metadata availability across studies restricted our ability to account for potential confounders beyond disease status, sex, BMI, MS treatment, and age. Microbial cell counts required for quantitative profiling were also unavailable, limiting our analyses to relative microbial abundances. Furthermore, sex-related differences in gut microbiota, while more pronounced in patients, appeared as non-significant trends in the controls. These results suggest that sex differences should be explored in larger cohorts of healthy controls to further understand the extent and variation in gut microbiota sex differences within health boundaries. Finally, as this work relied on 16S rRNA gene sequencing, future studies using whole-genome shotgun metagenomics and metabolomics will be essential to elucidate functional and mechanistic links between sex, gut microbiota, and MS.

Overall, despite the inherent challenges of microbiome research, this study provides a novel and integrative perspective on the interplay between sex, the gut microbiota, and MS. Results are readily accessible through a freely available, user-friendly web resource to facilitate further research. Sex-differential microbial composition may have the potential to influence MS pathogenesis, underscoring the need for further research to clarify their role in disease modulation.

## MATERIALS AND METHODS

Bioinformatics analyses were performed using a combination of bash scripting and R programming (v4.3.2). The developed code is available at https://github.com/IrSoler/cbl-metaanalysis-16SMS.

### Systematic review

A systematic review was conducted according to the Preferred Reporting Items for Systematic Reviews and Meta-Analyses (PRISMA) 2020 standards (72). Responses to the PRISMA abstract checklist are provided in the supplemental material. Literature screening was performed up to April 2025 using the European Nucleotide Archive (ENA) and the Sequence Read Archive (SRA) public repositories. Peer-reviewed publications with associated data were also searched for in PubMed. The following queries were applied: for ENA, multiple sclerosis microbiome; for SRA, ((multiple sclerosis*)* AND “human gut metagenome”[orgn:__txid408170]) AND bioproject_sra[filter]; and for PubMed (*“*multiple sclerosis”[All Fields] AND ((micro*[All Fields]) AND (human[All Fields] OR “homo sapiens”[All Fields]) AND (metagenom*[All Fields] OR metatranscript*[All Fields] OR 16S[All Fields]))).

All identified studies were individually reviewed. Studies were excluded if they met any of the following criteria: (i) methodology, studies that did not generate 16S metagenomic data; (ii) sample origin, studies not based on human fecal samples; (iii) disease examined, studies not focused on MS; (iv) experimental design, studies including only MS patients without control samples; (v) sex information, studies that did not record sex information; (vi) sample size, data sets with fewer than three individuals per condition and sex; and (vii) data availability, unavailability of FASTQ files and/or associated metadata.

FASTQ files and associated metadata from the selected studies were retrieved for primary bioinformatics analysis. Metadata nomenclature was standardized across all studies.

### Data processing for individual studies

Raw sequencing reads were filtered and trimmed using Cutadapt (73) (v4.6) to remove low-quality bases and/or reads, adapter sequences (when present), and undetermined nucleotides. Detailed quality control thresholds for each data set are provided in Table S3. Amplicon sequence variants (ASVs) were inferred using the DADA2 pipeline (74) (v1.30.0). Briefly, the workflow included dereplication, error rate learning, ASV inference, merging of paired-end reads (when applicable), chimera removal, statistical evaluation, construction of the ASV abundance matrix, and filtering of sequences with non-target lengths. Predefined parameters were applied, except for minFoldParentOverAbundance used for chimera detection, which was set to 8. Reads were discarded if they were not identified as ASVs, if forward and reverse reads could not be successfully merged (in paired-end data sets), if they were identified as chimeras, or if their length fell outside the expected range for the amplified region.

Taxonomic assignment was performed using the Genome Taxonomy Database release 220 (75). Taxonomic annotations for direct use in DADA2 were obtained from Dr. Claus Christophersen’s team-processed files (76). Each ASV was taxonomically classified at kingdom, phylum, class, order, family, and genus levels. ASVs that could not be confidently classified at a given taxonomic level were assigned as “unclassified” at the last reliably determined rank.

Quality assessment was performed to exclude unsuitable samples for downstream analyses. Samples were removed if they met any of the following exclusion criteria: (i) total read counts below 1,000, (ii) fewer than 30 distinct taxa identified, (iii) rarefaction curves not reaching a clear “plateau,” or (iv) evidence of contamination or poor processing based on the dominant taxonomic composition. The remaining samples constituted the filtered abundance matrices, which were the input for subsequent analyses that were performed at the genus level.

### Characterization of microbiota diversity

Inter- and intrastudy variability were characterized by quantifying within-sample diversity (alpha diversity and Shannon index) and between-sample dissimilarity (beta diversity and Bray–Curtis dissimilarity metric) with functions implemented in the phyloseq R package (77) (v1.46.0). Multivariate associations between microbiota composition and explanatory variables were assessed by distance-based redundancy analysis (dbRDA) with the vegan R package (78) (v2.6.8). Statistical significance was evaluated with the permutation-based ANOVA approach. Stepwise model building was performed to distinguish non-redundant sources of variation. The Benjamini–Hochberg (BH) method (79) was used to correct for multiple testing, considering significance when the FDR was <0.05.

### Individual differential abundance analysis

For each data set, previously filtered abundance matrices were subset to retain genera with a relative abundance of ≥0.01% in a minimum of 10% of the samples, reducing noise from rare or spurious taxa. Only taxa common to all data sets were selected to avoid data set-specific biases in taxonomic representation.

Filtered matrices were individually normalized using the variance stabilizing transformation method from the DESeq2 R package (80) (v1.42.1). Each data set was then subjected to differential abundance testing, evaluating the impact of sex in (i) the control group (“control females” vs “control males”) and (ii) the MS group (“MS females” vs “MS males”). For each comparison, the non-parametric Wilcoxon rank-sum test was used after confirming that no unwanted sources of variation were significant in the dbRDA analysis or the principal coordinate sample distribution.

For all studies, resulting *P* values were adjusted for multiple testing using the BH method (79), with significance set at an FDR of <0.05. To provide a quantitative estimate of the magnitude of observed differences, effect sizes were calculated using the wilcox_effsize function from the rstatix R package (81) (v0.7.2). Ninety-five percent confidence intervals for the effect sizes were estimated via bootstrap resampling with 1,000 iterations using the boot R package (82) (v1.3-28.1).

### Meta-analysis

Meta-analysis was performed for (i) MS females vs MS males and (ii) control females vs control males. For each comparison, individual effect sizes were integrated with the DerSimonian–Laird method (83) using the metafor R package (84). *P* values were adjusted with the BH method (79) (v4.8.0), considering significant results when the FDR was <0.05.

For each taxon and comparison, the heterogeneity indicators QE, QEp, SE, *τ*², *I*², and *H*² were calculated. Heterogeneity was also evaluated at the study level through influence and funnel plots. For influence plots, the metrics rstudent, dffits, cook.d, cov.r, tau2.del, QE.del, hat, and weight were calculated by sequentially removing one study and assessing the impact on the model parameters. For funnel plots, standard error and variance were used as measures of variability.

### Validation of meta-analysis findings

The validation data set (ID: PRJEB32762) comprises 16S rRNA gene sequencing and WGS metagenomic data. The 16S rRNA gene sequencing data were processed using the same pipeline as the data sets included in the integrative analysis. Detailed descriptions of the procedures can be found above in “Data processing for individual studies,” “Characterization of microbiota diversity,” and “Individual differential abundance analysis.” Raw WGS sequencing reads were preprocessed following the specificities detailed by the authors of the original publication (15). Taxonomic profiling was performed using MetaPhlAn (v4.0.1) with the ChocoPhlan (vJun23) reference database (85). Differential abundance analysis was performed at genus level, following the same statistical strategy described for the 16S rRNA gene sequencing data.

In this data set, a notable batch effect was identified associated with the enrollment group and the city where samples were collected. A combined batch variable was created by merging these two factors. Differential abundance testing was then performed using a blocked Wilcoxon rank-sum test with the wilcox_test function from the R coin package (86) (v1.4.3). The formula was determined as *y − x |* block, where *y* represents the abundance of the genus being tested; *x* represents the grouping variable defining the comparison of interest; and block corresponds to the combined batch factor.

Finally, differential abundance results were compared with those obtained from the meta-analysis considering statistical significance and the direction of change.

To evaluate whether the sex-based microbial differences identified in the meta-analysis were specific to the MS disease or reflected a broader sex effect, linear mixed models were fitted for the significant taxa in this 16S rRNA gene data set using the lme4 R package (v1.1.35.1). For each genus, we calculated the model: genus abundance − condition *×* sex *+* (1 | block), where condition and sex were included as fixed effects; their interaction term (condition:sex) was used to assess disease specificity of the sex effect; and block was included as a random effect to account for the combined batch factor. Control males were used as the reference group.

### Microbial association with MS features

Microbial associations with MS features were evaluated using the χ² goodness of fit test for categorical variables, the Wilcoxon rank-sum test for numerical vs binary categorical variables, the Kruskal–Wallis tests followed by Dunn’s *post hoc* test for numerical variables across multiple groups, and Spearman’s correlation for numerical variables. This framework was also applied to MS features to assess potential confounding. All tests were performed with functions from stats (87) (v4.3.2) and dunn.test (88) (v1.3.6) R packages. Significance was considered at *P* < 0.05 or FDR < 0.05 when multiple testing was applicable.

The same approach was used to examine associations among MS features and to identify potential confounding factors between sex and other metadata variables.

### Web tool

A web tool was developed to enable interactive exploration and visualization of the individual differential abundance and meta-analysis results (https://irsoler.shinyapps.io/metaanalisis_16S_MS/). This web-based application was built using the Shiny R package (89) (v1.9.1). It is hosted on shinyapps.io, a cloud-based platform for sharing interactive web applications built with Shiny.

## Supplementary Material

Reviewer comments

## Data Availability

The data sets analyzed in this work are publicly available: PRJNA684124 (11), PRJNA732670 (16), PRJEB99111 (6), PRJEB34168 (17), PRJNA565173 (18), PRJEB67783 (21) and PRJEB32762 (15). The bioinformatic code can be found at https://github.com/IrSoler/cbl-metaanalysis-16SMS, and the complete results can be explored at https://irsoler.shinyapps.io/metaanalisis_16S_MS.

## SUPPLEMENTAL MATERIAL

Supplemental material msystems.00416-26-s0001.docx Tables S1-S3 and Figures S1-S11.

PRISMA form msystems.00416-26-s0002.pdf PRISMA abstract checklist.

OPEN PEER REVIEW reviewer-comments.pdf An accounting of the reviewer comments and feedback.

ASM does not own the copyrights to Supplemental Material that may be linked to, or accessed through, an article. The authors have granted ASM a non-exclusive, world-wide license to publish the Supplemental Material files. Please contact the corresponding author directly for reuse.
