# Supplementary material for: Integrative multicohort analysis reveals consistent sex differences in gut microbiota of multiple sclerosis patients
Source: mSystems. 2026 Jul 22;11(8):e00416-26. doi: 10.1128/msystems.00416-26 (PMC13483414; doi:10.1128/msystems.00416-26)
Supplement: Supplementary file 2 [file reviewer-comments.pdf]

AMERICAN SOCIETY FOR MICROBIOLOGY | mSystems®

Integrative multi-cohort analysis reveals consistent sex differences in gut microbiota of multiple sclerosis patients

Irene Soler-Sáez, Cristina Galiana-Roselló, Rubén Grillo-Risco, Gwen Falony, Vanja Tepavčević, Sara Vieira Silva, and Francisco García-García

Corresponding Author(s): Francisco García-García, Fundacion de la Comunidad Valenciana Centro de Investigacion Principe Felipe

Review Timeline:

Submission Date:	April 15, 2026
Editorial Decision:	May 14, 2026
Revision Received:	June 9, 2026
Accepted:	July 3, 2026

Editor: Suleyman Yildirim

Reviewer(s): Disclosure of reviewer identity is with reference to reviewer comments included in decision letter(s). The following individuals involved in review of your submission have agreed to reveal their identity: Shirong Liu (Reviewer #2)

Transaction Report:

(Note: With the exception of the correction of typographical or spelling errors that could be a source of ambiguity, letters and reports are not edited. The original formatting of letters and referee reports may not be reflected in this compilation.)

DOI: https://doi.org/10.1128/msystems.00416-26

Re: mSystems00416-26 (**Integrative multi-cohort analysis reveals consistent sex differences in gut microbiota of multiple sclerosis patients**)

Dear Dr. Francisco García-García:

Thank you for the privilege of reviewing your work. Below you will find my comments, instructions from the mSystems editorial office, and the reviewer comments.

Please return the manuscript within 60 days; if you cannot complete the modification within this time period, please contact me. If you do not wish to modify the manuscript and prefer to submit it to another journal, notify me immediately so that the manuscript may be formally withdrawn from consideration by mSystems.

Revision Guidelines
To submit your modified manuscript, log into the submission site at https://msystems.msubmit.net/cgi-bin/main.plex. Go to Author Tasks and click the appropriate manuscript title to begin. The information you entered when you first submitted the paper will be displayed; update this as necessary. Note the following requirements:

• Upload point-by-point responses to the issues raised by the reviewers in a file named "Response to Reviewers," NOT in your cover letter.
• Upload a compare copy of the manuscript (without figures) as a "Marked-Up Manuscript" file.
• Upload a clean .DOC/.DOCX version of the revised manuscript and remove the previous version.
• Each figure must be uploaded as a separate, editable, high-resolution file (TIFF or EPS preferred), and any multipanel figures must be assembled into one file.
• Any supplemental material intended for posting by ASM should be uploaded with their legends separate from the main manuscript. You can combine all supplemental material into one file (preferred) or split it into a maximum of 10 files with all associated legends included.

For complete guidelines on revision requirements, see our Submission and Review Process webpage. Submission of a paper that does not conform to guidelines may delay acceptance of your manuscript.

Data availability: ASM policy requires that data be available to the public upon online posting of the article, so please verify all links to sequence records, if present, and make sure that each number retrieves the full record of the data. If a new accession number is not linked or a link is broken, provide mSystems production staff with the correct URL for the record. If the accession numbers for new data are not publicly accessible before the expected online posting of the article, publication may be delayed; please contact production staff (mSystems@asmusa.org) immediately with the expected release date.

Publication Fees: For information on publication fees and which article types are subject to charges, visit our website. If your manuscript is accepted for publication and any fees apply, you will be contacted separately about payment during the production process; please follow the instructions in that e-mail. Arrangements for payment must be made before your article is published.

ASM Membership: Corresponding authors may join or renew ASM membership to obtain discounts on publication fees. Need to upgrade your membership level? Please contact Customer Service at Service@asmusa.org.

The ASM Journals program strives for constant improvement in our submission and publication process. Please tell us how we can improve your experience by taking this quick Author Survey.

Thank you for submitting your paper to mSystems.

Sincerely,
Suleyman Yildirim
Editor
mSystems

Reviewer #2 (Comments for the Author):

This manuscript, "Integrative multi-cohort analysis reveals consistent sex differences in gut microbiota of multiple sclerosis patients," investigates important sex-specific differences in the gut microbiome of MS patients through analysis of six previously published independent cohorts. The authors identified sex-associated differences in gut microbial composition, including 11 microbial taxa showing significant sex-related alterations in MS, with nine enriched in females and two enriched in males. In

particular, they highlighted the female-enriched taxa Eggerthella and Eisenbergiella, which were associated with specific MS subtypes and greater disability. The authors further validated the identified genera using the independent iMSMS cohort and developed a freely accessible web-based platform providing comprehensive access to their dataset.
The findings are timely, meaningful, and of broad interest to the field. The manuscript is well written and should be considered for publication after addressing the following minor concern:
The Discussion section would benefit from additional commentary on why Eggerthella and Eisenbergiella were not identified as distinguishable MS-associated microbial signatures in the original iMSMS publication (PMID: 36113426). Clarifying the potential reasons for this discrepancy would strengthen the interpretation and translational relevance of the current findings.

Reviewer #3 (Comments for the Author):

In this manuscript, Soler-Sáez and colleagues conduct a meta-analysis of several 16S RNA studies in MS and find sex-associated taxa. The study included a large validation cohort. Despite significant dataset-to-dataset variation, the authors report that 11 taxa have sex associations (9 with females and 2 with males). In particular, Egghерthella lenta and Eisenbergiella Tayi were associated with MS subtypes and higher disability. Authors make a computational tool available for further exploration.

This is a well-written manuscript that describes a novel set of sex-specific associations with multiple sclerosis. The work exhibits high statistical rigor, and the conclusions drawn from the analysis are sound.
My main concern is that the authors compare F x M within each condition (MS or CTRL). However, a logistic model with covariates would provide greater power and enable the identification of sex-specific and interaction effects (sex * condition).
Also, authors mention that only 16S RNA sequencing was available for this analysis. However, according to the publication (PMID: 36113426), the validation cohort does include a metagenomic arm.

Reviewer #2 (Comments for the Author):

This manuscript, "Integrative multi-cohort analysis reveals consistent sex differences in gut microbiota of multiple sclerosis patients," investigates important sex-specific differences in the gut microbiome of MS patients through analysis of six previously published independent cohorts. The authors identified sex-associated differences in gut microbial composition, including 11 microbial taxa showing significant sex-related alterations in MS, with nine enriched in females and two enriched in males. In particular, they highlighted the female-enriched taxa Eggerthella and Eisenbergiella, which were associated with specific MS subtypes and greater disability. The authors further validated the identified genera using the independent iMSMS cohort and developed a freely accessible web-based platform providing comprehensive access to their dataset.

The findings are timely, meaningful, and of broad interest to the field. The manuscript is well written and should be considered for publication after addressing the following minor concern:
The Discussion section would benefit from additional commentary on why Eggerthella and Eisenbergiella were not identified as distinguishable MS-associated microbial signatures in the original iMSMS publication (PMID: 36113426). Clarifying the potential reasons for this discrepancy would strengthen the interpretation and translational relevance of the current findings.

Response: We thank the reviewer for the positive evaluation of our work and for pointing to this valuable suggestion. We added the following paragraph to the Discussion section contextualising our findings in relation to those from the iMSMS publication (lines: 449-458).

Of note, in the iMSMS publication (15) the authors compared the gut microbiome composition of MS patients and control individuals adjusting for sex among other covariates. They identified Eisenbergiella as an MS-associated taxon with higher abundance in progressive MS, consistent with our findings. In contrast, Eggerthella was not identified as significantly associated with MS. In our study, the abundance of Eggerthella appears to be primarily determined by sex rather than by disease condition itself, suggesting that its differential abundance may reflect a sex-dependent modulation exacerbated within the MS context rather than a direct disease-specific association. These findings highlight the complementary value of condition-stratified sex-associated analyses in identifying differential abundance patterns that may remain undetected in case-control study designs.

Reviewer #3 (Comments for the Author):

In this manuscript, Soler-Sáez and colleagues conduct a meta-analysis of several 16S RNA studies in MS and find sex-associated taxa. The study included a large validation cohort. Despite significant dataset-to-dataset variation, the authors report that 11 taxa have sex associations (9 with females and 2 with males). In particular, Egghertella lenta and Eisenbergiella Tayi were associated with MS subtypes and higher disability. Authors make a computational tool available for further exploration.

This is a well-written manuscript that describes a novel set of sex-specific associations with multiple sclerosis. The work exhibits high statistical rigor, and the conclusions drawn from the analysis are sound.

Response: We thank the reviewer for recognising the significance of this work. We appreciate the constructive comments and suggestions provided.

My main concern is that the authors compare F x M within each condition (MS or CTRL). However, a logistic model with covariates would provide greater power and enable the identification of sex-specific and interaction effects (sex * condition).

Response: We acknowledge that an interaction model offers greater statistical power to characterize sex and condition interaction effects. To address this, we applied linear mixed models, with batch included as a random effect to account for the variability associated with the recruitment group and site. Specifically, for the 11 taxa found to be significant in the MS females vs. MS males comparison in the meta-analysis, we fitted the following model in the iMSMS cohort, using control males as the reference group:

Genus abundance ~ Condition * Sex + (1 | batch)

This approach allowed us to evaluate whether the observed sex differences were i) specific to the MS condition, ii) more broadly attributable to sex independently of disease status (e.g. *Eggerthella* and *Flavonifractor*) or iii) presented a more complex pattern with the interaction of the two variables (e.g. *Eisenbergiella*). The results of this analysis are reported in lines 298-308, and the statistical approach is described in the Material and Methods section (lines: 631-638).

Also, authors mention that only 16S RNA sequencing was available for this analysis. However, according to the publication (PMID: 36113426), the validation cohort does include a metagenomic arm.

Response: We thank the reviewer for this observation. Our study was designed to analyse 16S data, given the greater availability of datasets to be evaluated. To benefit from the WGS data from the iMSMS cohort, we additionally processed this metagenomic dataset and performed the corresponding sex-based comparison (MS females vs. MS males) for the 11 taxa identified as significant in our meta-analysis. The majority of the findings derived from the WGS data were consistent with those obtained from the 16S rRNA gene analysis, further supporting the reproducibility of the identified sex-differential abundant taxa. These results have been incorporated into the manuscript in the Results section (lines: 290-296) and the Material and Methods section (lines: 614-618).

Re: mSystems00416-26R1 (**Integrative multi-cohort analysis reveals consistent sex differences in gut microbiota of multiple sclerosis patients**)

Dear Dr. Francisco García-García:

Your manuscript has been accepted, and I am forwarding it to the ASM production staff for publication. Your paper will first be checked to make sure all elements meet the technical requirements. ASM staff will contact you if anything needs to be revised before copyediting and production can begin. Otherwise, you will be notified when your proofs are ready to be viewed.

Data Availability: ASM policy requires that data be available to the public upon online posting of the article, so please verify all links to sequence records, if present, and make sure that each number retrieves the full record of the data. If a new accession number is not linked or a link is broken, provide production staff with the correct URL for the record. If the accession numbers for new data are not publicly accessible before the expected online posting of the article, publication may be delayed; please contact ASM production staff immediately with the expected release date.

Publication Fees: For information on publication fees and which article types have charges, please visit our website. We have partnered with Copyright Clearance Center (CCC) to collect author charges. If fees apply to your paper, you will receive a message from no-reply@copyright.com with further instructions. For questions related to paying charges through RightsLink, please contact CCC at ASM_Support@copyright.com or toll free at +1-877-622-5543. CCC makes every attempt to respond to all emails within 24 hours.

ASM Membership: Corresponding authors may join or renew ASM membership to obtain discounts on publication fees. Need to upgrade your membership level? Please contact Customer Service at Service@asmusa.org.

Embargo Policy: A press release may be issued as soon as the manuscript is posted on the mSystems Latest Articles webpage. The corresponding author will receive an email with the subject line "ASM Journals Author Services Notification" when the article is available online.

Cover Image Submissions: If you would like to submit a potential Cover Image, please email a file and a short legend to msystems@asmusa.org. Please note that we can only consider images that (i) the authors created or own and (ii) have not been previously published. By submitting, you agree that the image can be used under the same terms as the published article. Image File requirements: TIF/EPS, 7.5 inches wide by 8.25 inches tall (at least 2,250 pixels wide by 2,475 pixels tall), minimum 300 dpi resolution (600 dpi preferred), RGB, and no figure elements, e.g., arrows or panel labels. The legend should be a short description of the image, 1-2 sentences recommended. Please download and use this interactive template in Adobe to ensure that your proposed cover image meets our size requirements (https://journals.asm.org/pb-assets/pdf-text-excel-files/ASM-Interactive-Sizing-Cover-Template-1715689791.pdf).

Thank you for submitting your paper to mSystems.

Sincerely,
Suleyman Yildirim
Editor
mSystems
