# Supplementary material for: Integrative multicohort analysis reveals consistent sex differences in gut microbiota of multiple sclerosis patients
Source: mSystems. 2026 Jul 22;11(8):e00416-26. doi: 10.1128/msystems.00416-26 (PMC13483414; doi:10.1128/msystems.00416-26)
Supplement: Supplementary file 3 [file msystems.00416-26-s0001.docx]

## **TABLE OF CONTENTS**

| **Supplementary Table 1.** Within-dataset variability determined by distance-based redundancy analysis (dbRDA). | *2* |
| --- | --- |
| **Supplementary Table 2.** Linear mixed model results for the 11 taxa significant in the MS females vs. MS males meta-analysis, evaluated in the iMSMS validation cohort. | *3* |
| **Supplementary Table 3.** Overview of the preprocessing parameters by dataset. | *5* |
|  |  |
| **Supplementary Figure 1.** Sample distribution by dataset. | *6* |
| **Supplementary Figure 2.** Alpha diversity distribution across the six datasets included in the meta-analysis (A) by dataset and (B) by condition and sex. | *7* |
| **Supplementary Figure 3**. Sex-differential abundance patterns by individual datasets in (A) controls and (B) MS populations. | *8* |
| **Supplementary Figure 4.** Meta-analysis results for (A) Eggerthella and (B) Desulfovibrio when comparing MS females versus MS males (MS comparison). | *9* |
| **Supplementary Figure 5**. Meta-analysis results for (A) Eisenbergiella (B) Holdemania and (C) Lawsonibacter_A when comparing MS females versus MS males (MS comparison). | *10* |
| **Supplementary Figure 6.** Meta-analysis results for (A) Pseudoruminococcus (B) Scatomorpha and (C) UMGS1071 when comparing MS females versus MS males (MS comparison). | *11* |
| **Supplementary Figure 7.** Meta-analysis results for (A) Prevotella (B) Flavonifractor and (C) Catenibacterium when comparing MS females versus MS males (MS comparison). | *12* |
| **Supplementary Figure 8.** Alpha diversity distribution in the validation MS cohort (A) by recruitment site (B) enrollment group and (C) by condition and sex. | *13* |
| **Supplementary Figure 9.** Concordance of sex-differential abundance patterns in the MS comparison at 16S rRNA gene and WGS sequencing resolution. | *13* |
| **Supplementary Figure 10.** Normalized abundance of the validated genera in treated and untreated patients within the multiple sclerosis cohort of the validation dataset. | *14* |
| **Supplementary Figure 11.** Associations between sex and clinical and demographic variables in the MS cohort. | *15* |

| **Dataset** | **Samples** | **Significant variables**  **(% variance)** | **Not significant variables** |
| --- | --- | --- | --- |
| **PRJNA684124** | All samples | Sex (2.27%) | Condition, age |
|  | MS samples | None | Treatment |
| **PRJEB99111** | All samples | None | Condition, age, sex,  sequencing set,  center |
| **PRJNA732670** | All samples | Condition (3.1%) | BMI, age, sex |
|  | MS samples | None | Treatment |
| **PRJEB34168** | All samples | Condition (1.22%) | BMI, age, sex |
| **PRJNA565173** | All samples | BMI (3.71%),  condition (2.38%) | Age, sex |
| **PRJEB67783** | All samples | None | Condition, BMI, age,  sex |
|  | MS samples | None | Treatment, MS subtype |

**Supplementary Table 1. Within-dataset variability determined by distance-based redundancy analysis (dbRDA).** For each dataset, the table reports: (i) variables identified as significant in univariate models, together with the percentage of variance explained (in parentheses); and (ii) variables tested that were not statistically significant. The MS subcohort was evaluated only for MS-specific variables (treatment and MS subtype), when available. Statistical significance was defined as *p* < 0.05. *BMI, body mass index; MS, multiple sclerosis.*

| **Genus** | **Term** | **Estimate** | **std**  **error** | **stat** | **raw *p*** | **adjusted *p*** |
| --- | --- | --- | --- | --- | --- | --- |
| *Catenibacterium* | ConditionMS | -0.467 | 0.453 | -1.03 | 0.303 | 0.477 |
| *Desulfovibrio* | ConditionMS | 0.611 | 0.399 | 1.53 | 0.125 | 0.276 |
| ***Eggerthella*** | **ConditionMS** | **0.248** | **0.376** | **0.659** | **0.51** | **0.701** |
| ***Eisenbergiella*** | **ConditionMS** | **0.991** | **0.444** | **2.23** | **0.026** | **0.143** |
| ***Flavonifractor*** | **ConditionMS** | **0.556** | **0.442** | **1.26** | **0.209** | **0.382** |
| *Holdemania* | ConditionMS | -0.0565 | 0.363 | -0.156 | 0.876 | 0.876 |
| *Lawsonibacter_A* | ConditionMS | 0.516 | 0.279 | 1.85 | 0.064 | 0.237 |
| *Pseudoruminococcus* | ConditionMS | 0.623 | 0.389 | 1.6 | 0.11 | 0.276 |
| *Scatomorpha* | ConditionMS | 1.15 | 0.403 | 2.85 | 0.004 | 0.0489 |
| *UMGS1071* | ConditionMS | 0.112 | 0.422 | 0.266 | 0.79 | 0.869 |
| ***Prevotella*** | **ConditionMS** | **-0.206** | **0.448** | **-0.46** | **0.646** | **0.79** |
| *Catenibacterium* | SexFemale | 0.181 | 0.434 | 0.418 | 0.676 | 0.775 |
| *Desulfovibrio* | SexFemale | -0.109 | 0.382 | -0.286 | 0.775 | 0.775 |
| ***Eggerthella*** | **SexFemale** | **1.09** | **0.36** | **3.02** | **0.002** | **0.0143** |
| ***Eisenbergiella*** | **SexFemale** | **0.75** | **0.425** | **1.76** | **0.078** | **0.144** |
| ***Flavonifractor*** | **SexFemale** | **1.1** | **0.423** | **2.61** | **0.009** | **0.0256** |
| *Holdemania* | SexFemale | -0.111 | 0.347 | -0.32 | 0.749 | 0.775 |
| *Lawsonibacter_A* | SexFemale | 0.508 | 0.267 | 1.9 | 0.057 | 0.126 |
| *Pseudoruminococcus* | SexFemale | 0.977 | 0.373 | 2.62 | 0.008 | 0.0256 |
| *Scatomorpha* | SexFemale | 1.23 | 0.385 | 3.2 | 0.001 | 0.0143 |
| *UMGS1071* | SexFemale | 0.551 | 0.404 | 1.36 | 0.173 | 0.238 |
| ***Prevotella*** | **SexFemale** | **-0.698** | **0.429** | **-1.63** | **0.103** | **0.163** |
| *Catenibacterium* | ConditionMS:SexFemale | -0.339 | 0.626 | -0.542 | 0.588 | 0.74 |
| *Desulfovibrio* | ConditionMS:SexFemale | -0.284 | 0.551 | -0.516 | 0.606 | 0.74 |
| ***Eggerthella*** | **ConditionMS:SexFemale** | **0.297** | **0.519** | **0.571** | **0.568** | **0.74** |
| ***Eisenbergiella*** | **ConditionMS:SexFemale** | **0.886** | **0.614** | **1.44** | **0.149** | **0.74** |
| ***Flavonifractor*** | **ConditionMS:SexFemale** | **0.327** | **0.61** | **0.536** | **0.592** | **0.74** |
| *Holdemania* | ConditionMS:SexFemale | -0.0667 | 0.501 | -0.133 | 0.894 | 0.894 |
| *Lawsonibacter_A* | ConditionMS:SexFemale | -0.389 | 0.385 | -1.01 | 0.313 | 0.74 |
| *Pseudoruminococcus* | ConditionMS:SexFemale | -0.953 | 0.537 | -1.77 | 0.076 | 0.74 |
| *Scatomorpha* | ConditionMS:SexFemale | -0.695 | 0.556 | -1.25 | 0.212 | 0.74 |
| *UMGS1071* | ConditionMS:SexFemale | 0.301 | 0.583 | 0.517 | 0.605 | 0.74 |
| ***Prevotella*** | **ConditionMS:SexFemale** | **-0.174** | **0.619** | **-0.281** | **0.779** | **0.856** |

**Supplementary Table 2. Linear mixed model results for the 11 taxa significant in the MS females vs. MS males meta-analysis, evaluated in the iMSMS validation cohort**. Three terms are reported for each taxon: ConditionMS (effect of disease status), SexFemale (effect of sex), and ConditionMS:SexFemale (sex-by-condition interaction). Control males were set as the reference group. Bold entries indicate taxa of interest in the main text: *Eggerthella*, *Eisenbergiella*, *Flavonifractor*, and *Prevotella*.

| **Dataset** | **Type** | **Region** | **Technology** | **Original length (bp)** | **Trimming 5’ (bp)** | **Trimming 3’ (bp)** | **Min length (bp)** |
| --- | --- | --- | --- | --- | --- | --- | --- |
| **PRJNA684124** | Paired-end | V3-V4 | Illumina MiSeq | 300 | 10, 10 | 10, 50 | 125 |
| **PRJNA721421** | Paired-end | V4 | Illumina MiSeq | Variable | 10, 10 | 10, 10 | 125 |
| **PRJEB99111** | Single-end | V4 | Illumina MiSeq | 150 | 10 | 0 | 75 |
| **PRJNA748865** | Single-end | V2:V4-V6:V9 | Ion Torrent | Variable | trunQ = 25 | | 50 |
| **PRJNA889427** | Paired-end | V4 | Illumina MiSeq | 250 | 10, 10 | 0, 0 | 125 |
| **PRJNA732670** | Paired-end | V3-V4 | Illumina MiSeq | 300 | 10, 10 | 10, 30 | 125 |
| **PRJEB34168** | Paired-end | V4 | Illumina MiSeq | 300 | 20, 20 | 20, 40 | 125 |
| **PRJNA565173** | Paired-end | V3-V4 | Illumina MiSeq | 280 | 10, 10 | 20, 50 | 125 |
| **PRJEB67783** | Paired-end | V3-V4 | Illumina MiSeq | 300 | 20, 20 | 20, 50 | 125 |
| **PRJEB32762** | Single-end | V4 | Illumina MiSeq | 150 | 10 | 0 | 75 |

**Supplementary Table 3. Overview of the preprocessing parameters by dataset.** For each dataset, the following information is provided: dataset identifier, sequencing mode (paired-end or single-end), targeted 16S rRNA gene region (V), sequencing technology, original read length (in base pairs), trimming cutoffs applied at the 5′ and 3′ ends, and the minimum read length (in base pairs) retained after quality trimming.

**Supplementary Figure 1. Sample distribution by dataset.** Dotplot representation based on the first two principal coordinates based on Bray-Curtis distance, with samples colored by dataset. Marked differences in taxonomic composition were observed across studies. In PRJNA748865, approximately half the number of distinct genera were identified compared with other datasets, and a large proportion of reads were classified as *unclassified Bacteria*. In PRJNA721421 and PRJNA889427, an unexpectedly high relative abundance of *Blautia* was detected, together with a lower-than-expected abundance (or absence) of typically dominant genera such as *Bacteroides*, *Phocaeicola*, and *Prevotella*.

**Supplementary Figure 2. Alpha diversity distribution across the six datasets included in the meta-analysis (A) by dataset and (B) by condition and sex.** Asterisks (*) indicate pairwise significant differences with adjusted p-values < 0.05 from Dunn’s post hoc test (after p-value < 0.05 in Kruskal-Wallis test). *MS: multiple sclerosis.*

**Supplementary Figure 3. Sex-differential abundance patterns by individual datasets in (A) controls and (B) MS populations.** Columns (datasets) and rows (genera) clustered based on hierarchical clustering. The color indicates the direction of the effect size (red: higher abundance in females, blue: higher abundance in males). Color intensity corresponds to the magnitude of the effect size. White dots: *p* < 0.05. *MS: multiple sclerosis.*

**Supplementary Figure 4. Meta-analysis results for (A) *Eggerthella* and (B) *Desulfovibrio* when comparing MS females versus MS males (MS comparison).** From left to right: (i) Forest plots: Each point represents the effect size from an individual dataset, with horizontal lines indicating the 95% confidence intervals. The diamond at the bottom represents the combined effect size and its confidence interval. Positive values indicate higher abundance in MS females; negative values indicate higher abundance in MS males. (ii) Funnel plots: effect sizes (X-axis) compared to precision estimates (Y-axis). Each point represents an individual dataset effect size plotted against its standard error (top left), sample variance (top right), inverse standard error (bottom left), and inverse sampling variance (bottom right). The white triangle shows the 95% confidence region under the null hypothesis of no bias. (iii) Influence plots: assessment of each study's influence on the combined effect size using multiple diagnostic metrics. Study ID numbers are ordered based on their position in the forest plot (e.g., PRJEB34168 corresponds to study 1). Detailed description of the metric is defined in the Materials and Methods section. Red dots: influential studies; grey dots: non influential studies. *CI: Confidence Interval; DL: DerSimonian-Laird; ID: Identifier.*

**Supplementary Figure 5. Meta-analysis results for (A) *Eisenbergiella* (B) *Holdemania* and (C) *Lawsonibacter_A* when comparing MS females versus MS males (MS comparison).** From left to right: (i) Forest plots: Each point represents the effect size from an individual dataset, with horizontal lines indicating the 95% confidence intervals. The diamond at the bottom represents the combined effect size and its confidence interval. Positive values indicate higher abundance in MS females; negative values indicate higher abundance in MS males. (ii) Funnel plots: effect sizes (Xaxis) compared to precision estimates (Y-axis). Each point represents an individual dataset effect size plotted against its standard error (top left), sample variance (top right), inverse standard error (bottom left), and inverse sampling variance (bottom right). The white triangle shows the 95% confidence region under the null hypothesis of no bias. (iii) Influence plots: assessment of each study's influence on the combined effect size using multiple diagnostic metrics. Study ID numbers are ordered based on their position in the forest plot (e.g., PRJEB34168 corresponds to study 1). Detailed description of the metric is defined in the Materials and Methods section. Red dots: influential studies; grey dots: non influential studies. *CI: Confidence Interval; DL: DerSimonian-Laird; ID: Identifier.*

**Supplementary Figure 6. Meta-analysis results for (A) *Pseudoruminococcus* (B) *Scatomorpha* and (C) *UMGS1071* when comparing MS females versus MS males (MS comparison).** From left to right: (i) Forest plots: Each point represents the effect size from an individual dataset, with horizontal lines indicating the 95% confidence intervals. The diamond at the bottom represents the combined effect size and its confidence interval. Positive values indicate higher abundance in MS females; negative values indicate higher abundance in MS males. (ii) Funnel plots: effect sizes (X-axis) compared to precision estimates (Y-axis). Each point represents an individual dataset effect size plotted against its standard error (top left), sample variance (top right), inverse standard error (bottom left), and inverse sampling variance (bottom right). The white triangle shows the 95% confidence region under the null hypothesis of no bias. (iii) Influence plots: assessment of each study's influence on the combined effect size using multiple diagnostic metrics. Study ID numbers are ordered based on their position in the forest plot (e.g., PRJEB34168 corresponds to study 1). Detailed description of the metric is defined in the Materials and Methods section. Red dots: influential studies; grey dots: non influential studies. *CI: Confidence Interval; DL: DerSimonianLaird; ID: Identifier.*

**Supplementary Figure 7. Meta-analysis results for (A) *Prevotella* (B) *Flavonifractor* and (C) *Catenibacterium* when comparing MS females versus MS males (MS comparison).** From left to right: (i) Forest plots: Each point represents the effect size from an individual dataset, with horizontal lines indicating the 95% confidence intervals. The diamond at the bottom represents the combined effect size and its confidence interval. Positive values indicate higher abundance in MS females; negative values indicate higher abundance in MS males. (ii) Funnel plots: effect sizes (X-axis) compared to precision estimates (Y-axis). Each point represents an individual dataset effect size plotted against its standard error (top left), sample variance (top right), inverse standard error (bottom left), and inverse sampling variance (bottom right). The white triangle shows the 95% confidence region under the null hypothesis of no bias. (iii) Influence plots: assessment of each study's influence on the combined effect size using multiple diagnostic metrics. Study ID numbers are ordered based on their position in the forest plot (e.g., PRJEB34168 corresponds to study 1). Detailed description of the metric is defined in the Materials and Methods section. Red dots: influential studies; grey dots: non influential studies. *CI: Confidence Interval; DL: DerSimonian Laird; ID: Identifier.*

**Supplementary Figure 8. Alpha diversity distribution in the validation MS cohort (A) by recruitment site (B) enrollment group and (C) by condition and sex.** Asterisks (*) indicate pairwise significant differences with adjusted p-values < 0.05 from Dunn’s post hoc test (after p-value < 0.05 in Kruskal–Wallis test). *MS: multiple sclerosis.*

**Supplementary Figure 9. Concordance of sex-differential abundance patterns in the MS comparison at 16S rRNA gene and WGS sequencing resolution.** Direction of effect (comparison MS females vs. MS males) for taxa identified as significant in the meta-analysis, compared with corresponding results in the validation cohort with 16S rRNA gene and WGS sequencing. White dot: adjusted p < 0.05. *MS: multiple sclerosis, WGS: whole genome sequencing*.

**Supplementary Figure 10. Normalized abundance of the validated genera in treated and untreated patients within the multiple sclerosis cohort of the validation dataset.** Wilcoxon test adjusted p-values: *Eisenbergiella* (0.91), *Eggerthella* (0.90), *Flavonifractor* (0.89), and *Prevotella* (0.91). *VST: variance stabilizing transformation.*

**Supplementary Figure 11. Associations between sex and clinical and demographic variables in the MS cohort.** Panels A–C show comparisons between females and males for: (A) age, (B) disease duration and (C) BMI, assessed using the Wilcoxon test. Panels D–F display associations between sex and categorical variables: (D) MS subtype and (E) disability status classified as low vs. high disability (EDSS > 4), evaluated using the chi-square test. Adjusted *p*: age (0.47), disease duration (0.22), BMI (0.12), MS subtype (0.07), disability (0.42). *BMI: body mass index; MS: multiple sclerosis.*
